# Comparisons of an Open-Ended vs. Forced-Choice ‘Mind Reading’ Task: Implications for Measuring Perspective-Taking and Emotion Recognition

**DOI:** 10.1371/journal.pone.0093653

**Published:** 2014-12-04

**Authors:** Tracy G. Cassels, Susan A. J. Birch

**Affiliations:** Department of Psychology, The University of British Columbia, Vancouver, British Columbia, Canada; George Mason University/Krasnow Institute for Advanced Study, United States of America

## Abstract

Perspective-taking and emotion recognition are essential for successful social development and have been the focus of developmental research for many years. Although the two abilities often overlap, they are distinct and our understanding of these abilities critically rests upon the efficacy of existing measures. Lessons from the literature differentiating recall versus recognition memory tasks led us to hypothesize that an open-ended emotion recognition measure would be less reliant on compensatory strategies and hence a more specific measure of emotion recognition abilities than a forced-choice task. To this end, we compared an open-ended version of the *Reading the Mind in the Eyes Task* with the original forced-choice version in two studies: 118 typically-developing 4- to 8-year-olds (Study 1) and 139 5- to 12-year-olds; 85 typically-developing and 54 with learning disorders (Study 2). We found that the open-ended version of the task was a better predictor of empathy and more reliably discriminated typically-developing children from those with learning disorders. As a whole, the results suggest that the open-ended version is a more sensitive measure of emotion recognition specifically.

## Introduction

Recognizing, reasoning about and responding to the mental and emotional states of others are fundamental aspects of social interaction and development. Deficits in these abilities have been linked to a number of social problems found in clinical populations, such as those with depression, schizophrenia, Autistic Spectrum Disorders (ASDs), and children with conduct disorder [1]–[6]. However, even individual differences in these perspective-taking abilities within non-clinical populations are related to social adjustment, moral development, and academic competence [7]–[10], amongst others. Herein, we use the term ‘perspective-taking’ to refer to the broad set of abilities involved in reasoning about the mental states of others–including inferences about either one’s cognitive mental states or one’s affective mental states. We use the terms ‘emotion recognition’ and ‘affective perspective-taking’ when referring to one’s ability to infer affective mental states specifically (e.g., anger, sadness, joy) and ‘cognitive perspective-taking’ when referring to one’s ability to infer cognitive mental states (e.g., knowledge, thoughts, beliefs). Clearly there are many important reasons to study perspective-taking, and naturally, the utility of perspective-taking research critically rests upon how well the available measurement tools capture the dimensions of interest.

Many of the measures that are utilized in perspective-taking research today are forced-choice in nature. That is, individuals are presented with a limited set of pre-generated options for answers and asked to select the correct one. This is in contrast to open-ended tasks that involve posing a question (e.g., “What is he thinking?” or “Why is he thinking that?”) and allowing the respondent to spontaneously generate their own answer. The differences between the two task types may seem trivial, yet there are multiple reasons to believe this is an especially important question for investigation. In many ways the arguments parallel those in the literature for distinguishing between recall and recognition memory. A vast body of work has repeatedly shown that open-ended (i.e., free recall) memory tasks tap distinct processes from those elicited by forced-choice (i.e., recognition) memory tasks, with the latter usually being less sensitive for detecting subtle, underlying memory deficits [11]–[12]. Notably, forced-choice memory tasks provide response options that may serve as cues to elicit an answer that might not have otherwise come to mind and moreover allow for alternative strategies for solving the task that do not tap memory processes, such as ‘process-of-elimination’ (e.g., ‘I know it’s not A or B it must be C’).

Following similar logic, a forced-choice perspective-taking task may recruit less ‘perspective-taking specific’ processes than an open-ended format. Moreover, many of the populations that are studied with these measurement tools (e.g., individuals with ASDs) are the very individuals who are most apt to employ compensatory strategies (because of their deficits). Indeed, one study found no *behavioural* differences in a sample of college adults in their performance on a forced-choice emotion recognition task based on a median-split of psychopathic tendencies [13]. However, the researchers found differences in the groups’ neurological activation: The group with fewer psychopathic tendencies utilized brain regions involved in affective processing, whereas those with higher tendencies relied on regions associated with perception and cognition. One explanation of these findings is that at least some individuals are, under certain conditions (e.g., in a forced-choice task), able to circumvent their deficits by using cognitive strategies to ‘solve’ the task.

A second reason for favoring open-ended perspective-taking tasks to their forced-choice counterparts is that open-ended tasks more closely approximate ‘real-world’ emotion recognition. In naturalistic circumstances, one must *spontaneously* identify what another person is feeling or thinking; one does not have the benefit of guessing from a small set of pre-generated responses. Consequently, forced-choice tasks may be less ecologically valid than tasks that require one to *generate* mental state terms without aide.

Yet another reason to favour open-ended perspective-taking tasks, especially in children and certain clinical populations, stems from the tendency of forced-choice tasks to rely on a specific threshold of verbal proficiency or vocabulary knowledge. One of the primary processes linked to forms of perspective-taking is language [14], so much so that often a certain level of verbal proficiency is necessary to ‘pass’ these tasks [14]–[15]. Given the strong relationship between perspective-taking and verbal ability, it is probable that many of the tasks currently used to assess perspective-taking are simultaneously (and perhaps unnecessarily) tapping verbal ability. Worse still is the possibility that forced-choice perspective-taking tasks will not *accurately* assess perspective-taking abilities when verbal prerequisites are not met. Although it has sometimes been suggested that open-ended response options are more reliant upon verbal ability or vocabulary knowledge than forced-choice options, this depends greatly on the nature of the task and the manner in which responses are coded. If an individual must provide a very specific answer (e.g., ‘worried’), vocabulary knowledge will undoubtedly play a large role. However, if the task and coding is such that individuals can provide the ‘gist’ of the answer (e.g., ‘nervous’) then it opens the door for those with more limited vocabularies to be qualitatively correct.

One commonly-used measure of perspective-taking was developed by Simon Baron-Cohen and his colleagues and is known as the ‘Reading the Mind in the Eyes’ task (henceforth ‘*ET*’) [1]–[2]. In the task, participants are presented with several photographs of the eye-region and asked to choose which of four words best describes the person’s mental states. This was conceived as a measure of “how well the participant can put himself into the mind of the other person, and ‘tune in’ to their mental state” based on minimal cues [2, p.241]. The *ET* has proved effective in discriminating adults with Asperger’s syndrome or high-functioning autism (of normal intelligence) from controls [1]–[2]. In addition to work with those with ASDs, the *ET* has been used to examine perspective-taking deficits in other clinical populations, such as those with schizophrenia [4], patients who have had amygdalectomy or prefrontal cortical lesions [16], and depression [5]. A children’s version of the *ET* was developed by simplifying the response options and reducing the number of items and has been shown to discriminate between children with ASDs and those without [3].

The *ET* has been conceived as a measure of perspective-taking that encompasses both cognitive and affective components, although some researchers have utilized the *ET* as solely a cognitive perspective-taking task [6], [17] whereas others have utilized it solely as an emotion recognition task [18]–[20]. Its efficacy as an emotion recognition task per se is questionable given that it has never been validated as such and has failed to detect deficits where previous research suggests they should exist. For example, the *ET* was used to examine emotion recognition in relation to psychopathic tendencies in adults, revealing no deficits [18], [21], contrary to findings with adults [22] and children with related tendencies [23] that did not rely on the *ET* as the measure of emotion recognition. A potential reason for this discrepancy is that the forced-choice nature of the *ET* allows for the subjects’ cognitive skills to compensate for any deficits in their emotional ones. For these aforementioned reasons, we see considerable merit in examining the *ET’s* ability to measure emotion recognition per se and perspective-taking abilities more generally in comparison to an open-ended, or generative, version of the same task.

Undoubtedly, researchers have recognized the limitations of forced-choice emotion recognition tasks before, with some researchers using open-ended emotion recognition tasks with great success, even in preschool populations [24]. Nonetheless, presumably because of the ease of implementation, a tendency to rely on measures that have been previously used, and because much of the groundwork has not been laid for administering and coding open-ended versions, many researchers continue to use forced-choice tasks exclusively. Even though practicality is of obvious methodological import, measurement accuracy is arguably of greater importance and we saw considerable merit in creating a coding scheme for, and testing the efficacy of, an open-ended format to assess how response format changes what is being measured.

Although there are other emotion recognition tasks available for use with children, we have chosen the *ET* for a number of reasons. First and foremost, its widespread popularity allows comparisons to be made with other research. In addition, many of the emotion recognition measures used with children utilize stories or other descriptions to elicit a response [25]–[28] requiring considerable verbal ability or vocabulary knowledge, a factor we aimed to minimize.

The purpose of the present research was threefold: (1) to compare and contrast children’s performance on the standard (i.e., forced-choice) *ET* with a *generative* (i.e., open-ended) version of the same task, (2) to determine the convergent and discriminant validity of our open-ended version and coding scheme as both a perspective taking measure and an emotion recognition measure by examining the relationships of the two versions to verbal ability, empathy (believed to rely most on affective aspects of perspective-taking), and another widely-used measure of perspective-taking–*Happé’s Strange Stories* (believed to rely most on cognitive aspects of perspective-taking) and (3) to test how the two versions compare in predicting group membership between a sample of typically-developing children and a clinical sample (i.e., a learning disordered-sample known to have deficits in social-emotional skills and verbal ability). These goals were examined over two separate studies.

The face validity of the *ET* suggests it can be used as an *emotion recognition* task–it is, after all, a task that shows a particular expression in the eye-region of a person’s face and participants are asked ‘What is this person feeling?’. We hypothesized that an open-ended version should be an even more specific and sensitive measure of *emotion recognition* per se than the *ET* because it does not allow for cognitive strategies such as process of elimination and is stripped of its forced-choice response options that sometimes include cognitive terms such as ‘thinking of something’. In line with this, we predicted the open-ended version would be a) less related to *Happé’s Strange Stories* than the forced-choice *ET*, b) more related to empathic tendencies than the forced-choice *ET*, and c) a better predictor for discriminating typically-developing populations from atypically-developing populations with affective perspective-taking deficits.

## Method: Study 1

Study 1 was designed to compare the two task types in their ability to predict dispositional empathy and provide convergent evidence that an open-ended or generative version of the *ET* (henceforth ‘*GET*’) is a useful measure of *emotion recognition*. Given the abundance of evidence showing a positive relationship between emotion recognition and empathy– the ability and tendency to understand and respond to another person’s emotional state [29]–[31]–a childhood measure of empathy was selected to compare the affective nature of the *GET* and the *ET*.

### Ethics Statement

All children provided verbal assent and parents provided written consent prior to testing. The study was approved by the Behavioural and Research Ethics Board of the University of British Columbia.

### Participants

One hundred and eighteen participants were recruited from after-school care programs in Vancouver, Canada. Participants ranged in age from 4 to 8 years (72 boys; mean age = 5.84 years, SD* = *.97 years). An additional 16 participants (11.9% of the full sample) were excluded due to their failure to complete either the measure of dispositional empathy (9) or the *ET* or *GET* (7). In the opinion of the research assistants running these children, the failure to complete the dispositional empathy measure was most often due to failure to understand the questions whereas failure to complete either the *ET* or *GET* was more often due to parental interruption. Notably, if children found the *ET* or *GET* difficult and gave “Don’t know” as answers, those responses were included in the analyses.

### Measures

#### Reading the Mind in the Eyes, Children’s Version (ET) [3]

This task requires participants to choose the most appropriate term to describe the thoughts or feelings of others based upon still photographs of the eye-region of the face. Participants choose from four possible terms that researchers read aloud. For example, item 3 has the options of ‘friendly’, ‘surprised’, ‘sad’, and ‘worried’ (correct answer is ‘friendly’). See Appendix S1. Regardless of item type children are always asked, “What is this person feeling?”. A full statistical analysis of the *ET* (utilizing a subset of the data herein) was conducted using Confirmatory Factor Analysis (CFA) [28]. CFA revealed that the *ET* was best represented by one factor. Half of the participants completed this version of the Eyes task whereas the other half completed the Generative version.

#### Generative Eyes Task (GET) [28]

This task was created using the stimuli from the standard task. It is identical except that it requires participants to generate their own answer when asked “What is this person feeling?”. Answers were coded as correct if the participants’ answer matched the emotional category (i.e., positive, neutral, negative, or hostile) of the correct response from the forced-choice format. See Appendix S1 for the coding scheme and examples. Given it has been found that a two-factor structure (factor one representing items with an emotion valence and factor two representing items that are emotionally neutral) better fits the *GET* [28], the results for both the full version of the *GET and* the results for the 19-item *GET* that includes only the emotionally-valenced items (i.e., neutral items excluded) were included herein. The 19-item and full version *GET* were highly correlated, *r = *.790, *p<*.001.

#### Bryant Index of Empathy [32]

This is a 22-item self-report measure of dispositional empathy, validated for ages 5 through 13. The measure was presented verbally and in question form (instead of statement form). For example, the item “People who kiss and hug in public are silly” became “Do you think that people who kiss and hug in public are silly?” Options for answers are “True” or “Not true”, although as the measure was administered verbally in question form, we accepted “Yes” and “No” as answers. The measure provides one total score for dispositional empathy, and many items include assessment of the perspective-taking elements of empathy. The measure has good test-retest reliability (ranging from *r* = .74 to .81) and convergent and discriminant validity, as assessed using other measures of empathy (convergent) and reading-achievement scores (discriminant) [32]. In our sample, the alpha was .58, in line with the reported .54 for the 5–6 year-old group in the validation study.

### Procedure

All participants took part at their after-school care program. Participants were first given the *Bryant Index of Empathy* followed by either the *GET* or *ET*.

## Results: Study 1

### Preliminary Analyses

In the final sample, 65 participants completed the *ET* and 53 completed the *GET.* Independent samples t-tests were conducted to ensure that there were no differences between the two groups on gender and age, both non-significant (*p’s>*.30). Additionally, we wanted to ensure that there were no empathy differences between the two groups, and thus an independent samples t-test was conducted with dispositional empathy as the dependent variable. This too was non-significant, *t(116)* = .097, *p = *.923, *Cohen’s d = *.018, and thus any relationship with the Eyes tasks cannot be due to inherent group differences on empathy.

### Eyes Tasks Analyses

Scores on the two versions of the Eyes task were compared between groups using proportion correct. Participants who completed the *ET* (M* = *49.36%; range = 10.71% to 78.57%) performed much better than those who completed the *GET* (M = 29.32%; range = 7.14% to 46.43%). For the 19-item *GET* (M = 34.26%; range = 10.53% to 57.89%), results showed no significant differences in scores between the two versions, suggesting that the valenced items on the *GET* and the full version of the *ET* were equally difficult. See Table 1 for a full comparison. Importantly, the distributions on the two tasks were very similar, with neither demonstrating a ceiling or floor effect and both showed a relatively-normal distribution.

**Table 1:** Comparisons between the ET and the GET in the typically-developing samples from studies 1 and 2.

	Study 1	Study 2		
	Full Sample	Younger	Older	Full Sample
Mean Difference (full)	t(114) = 8.91^a,^***	t(47) = 6.333***	t(36) = 7.914***	t(84) = 9.857***
ET Mean (%)	49.36%	59.97%	69.69%	64.20%
GET Mean (%)	29.32%	43.01%	45.56%	44.11%
Effect Size	Cohen’s d = 1.67	Cohen’s d = 1.85	Cohen’s d = 2.64	Cohen’s d = 2.15
Mean Difference (shortened GET)	t(114) = .938	t(47) = 4.279***	t(36) = 7.891***	t(84) = 7.976***
ET Mean (%)	49.36%	59.97%	69.69%	64.20%
GET Mean (%)	34.36%	46.46%	44.05%	45.41%
Correlation	Cohen’s d = .18	Cohen’s d = 1.25	Cohen’s d = 2.63	Cohen’s d = 1.74

Note: ^a^Levene’s Test for Equality of Variances was significant, F(65,56) = 4.23, p = .042, and thus a case 4 t-test was used; ***p<.001; ET = Original Eyes Task; GET = Generative Eyes Task.

Age was found to be highly correlated with performance on the *ET*, *r = *.45, *p<*.001, with older children performing better, but showed no relationship to the *GET*, *r = *.06, *n.s.* [*r = *–.19, *n.s.*; 19-item *GET*]. Strictly speaking, no gender differences were identified for either the *ET* or *GET*, however, there was a trend on the *GET* favoring females, *t(51)* = 1.88, *p = *.065, *Cohen’s d = *.51 [*t(51)* = 1.92, *p = *.061, *Cohen’s d = *.54; 19-item *GET*].

### Empathy and the Eyes Tasks

To test the relationships between dispositional empathy and both the *GET* and *ET*, zero-order correlations were utilized. Dispositional empathy was unrelated to scores on the *ET*, *r = *.090, *n.s.*, but was significantly related to scores on the *GET*, *r = *.285, *p_1-tail_ = *.019 [*r = *.205, *p_1-tail_ = *.070; 19-item *GET*]. When Spearman’s correction for attenuation [33] was applied, the correlation between the *Bryant* and the *ET* rose to *r’* = .146, *n.s.*, and that for the *GET* rose to *r’ = *.614, *p<*.01 [*r’* = .381, *p<*.01; 19-item *GET*]. When the two correlations corrected for attenuation (between the *Bryant* and the *ET* and the *Bryant* and the *GET*) were compared, they were significantly different, *z = *2.96, *p_1-tail_ = *.002 [*z = *1.32, *p_1-tail_ = *.093; 19-item *GET*].

## Discussion: Study 1

The primary goals of the current study were to a) examine the reliability and performance of both the *GET* and *ET*, and b) provide convergent validity for the *GET* as an emotion recognition measure. With respect to the first goal, though the alpha of the full *GET* was low, the removal of neutral items increased the reliability and placed it on par with other measures for younger children, including the *ET* and the *Bryant Index of Empathy*. Though the average score for the *ET* was significantly greater than that for the *GET*, suggesting the *ET* is an easier task for this young age-group, that was not the case when the neutral items were removed from the *GET*.

We provided convergent validity for the *GET* as a measure of emotion recognition by showing that it was positively and significantly related to dispositional empathy, a relationship that was not present with the *ET*. With performance on the *GET* accounting for approximately 8% of the variance in empathy, this is similar in magnitude to previous studies examining the relationship between affective perspective-taking and empathy which have been in the range of 10% to 12% [29], [34]. Though one might expect a greater relationship between the valenced items of the *GET* and empathy, it is probable that the ability to also identify neutrality is important in understanding other people’s emotional states and thus the better one is at that, *in addition to identifying valenced emotions*, the more empathic one may be.

In sum, these results provide some convergent validity for the *GET* as an emotion recognition task and suggest that the *GET* is a more sensitive and specific measure of affective components of perspective-taking than the *ET*.

## Methods: Study 2

Study 2 was designed to provide discriminant validity for the *GET* by comparing performance on shortened versions of both the *GET* and *ET* to verbal ability and a measure of cognitive perspective-taking. A shortened version was utilized in order to provide within-subjects analyses with each participant completing half of the stimuli in open-ended form (i.e. the *GET*) and half as forced-choice (i.e., the *ET*). Furthermore, the original development of the *ET* was driven by the need to identify *differences* between clinical and typically-developing populations. Although much of the work on deficits in social functioning has targeted individuals with ASDs, there are other groups (such as those with learning disorders) who have demonstrated social deficits without more global cognitive impairment. In addition to comparing the short forms of the *ET* and *GET*, Study 2 examined the utility of these short forms in a clinical sample of children with learning disorders with known verbal and social-emotional deficits.

The choice of a learning-disordered sample stems in part from research on individuals with learning disorders that has found impairment in identifying emotional states relative to non-learning disordered individuals, even in adulthood [35]. Importantly, the diagnosis of specific learning disorders precludes a diagnosis of widespread cognitive disability [36], whereas a diagnosis of an ASD, for example, is often accompanied by clinical levels of cognitive impairment. Given the presence of both verbal and emotion recognition deficits in a learning-disordered population and the absence of general cognitive impairments, a sample of learning disordered individuals was deemed an appropriate clinical sample to test the feasibility of the *GET* for use with an atypically-developing population and to test the *GET* versus the *ET*’s power to predict group membership. A task that is purported to measure abilities associated with social functioning should discriminate any group with such deficits from their typically-developing counterparts. Given the presence of verbal deficits, we can compare how these tasks perform when controlling for verbal ability.

To these ends, children from the local area as well as children from a local school for those with learning disorders involving social-emotional deficits were recruited to take part in the study. The *ET*, *GET*, and a measure of verbal ability were administered and scores were compared to the typically-developing students to determine if there were significant differences between the two groups on each measure. We hypothesized that scores on the *ET* and *GET* would be lower than in a typically-developing group, in line with the work on emotion recognition deficits in those with learning disorders. We also expected that the *GET* would be a better predictor of group membership (typically-developing vs. those with learning disorders) than the *ET*.

### Ethics Statement

All children provided verbal assent and parents provided written consent. The study was approved by the Behavioural and Research Ethics Board of the University of British Columbia.

### Participants

Eighty-five typically-developing children (47 boys) from Vancouver, Canada participated in the K.I.D. Studies Centre at the University of British Columbia. The children ranged in age from 5- to 12-years (M = 8.18 years, SD = 1.60 years). Data from seven additional children were excluded due to the participants’ low proficiency in English because English was not their native language (4) or failure to complete the tasks (3). As the children in Study 1 were younger, the current sample was analyzed for children up to 8 years of age (M = 6.97 yrs; SD = .56 yrs) (n = 48; 26 boys) and children older than 8 (M = 9.75 yrs; SD = 1.04 yrs) (n = 37; 21 boys).

Additionally, fifty-four students (6- to 13-years old; M_age_ = 11.22 years, SD = 1.68 years; 29 boys) from a local school for children with learning disorders were recruited. For inclusion children had to have average intelligence, but show marked deficits in academic and social-emotional processing as assessed by school officials. In the current study, no participant was rated as having ‘no deficit’, and the participants with the *lowest* level of social weaknesses were rated as having “mild-moderate” deficits (n = 7), with the majority being rated as having more severe social deficits. Thus all participants met the criteria of having a social skills deficit. In addition, to be included they must not have had any brain injuries or severe behavioural problems.

### Measures

#### Reading the Mind in the Eyes, Children’s Version (ET) [3]

The *ET* is the same as used in Study 1. In the present study, however, only half of the stimuli were used so that the other 14 items could be administered in the generative version of the task. In the discriminant validity analyses, the same stimuli sets were used across all participants; however, for the group comparison, which stimuli set was used for the *ET* versus the *GET* was counterbalanced.

#### Generative Eyes Task (GET) [28]

The *GET* is the same as used in Study 1, but like the *ET*, only half of the stimuli were used. As the *GET* is best represented by two factors [28], the results are given using the full version (i.e., 14 items) and a 10-item version representative of the emotionally-valenced items for the comparisons in the typically-developing participants. Only the full version is used in the group comparisons analyses as the items used for the *ET* and *GET* were counterbalanced.

#### Happé’s Strange Stories [37]

This task involves answering questions about the non-literal statements made by characters in short stories and was completed only by the typically-developing students (to make comparisons to the *ET* and *GET*). Participants are asked two questions for each story: “Is what the person said true?” and “Why did they say it?” Correctly answering these questions requires that the participant understands the character’s mental states. Answers are coded based on how well the child’s answer reflects an awareness of the mental state behind the statement. The 12 different story types include: lie, white lie, joke, pretend, misunderstanding, persuade, appearance/reality, figure of speech, sarcasm, forget, contrary emotions, and double bluff. Only one of the stories pertains to emotional states, and it involves reasoning about the conditions that lead to contrary emotions; it does not involve emotion recognition, which is the primary interest in the *GET*. To reduce the burden on participants, only one of each type of story was used (12 of the original 24). The author of the original measure and members of her lab have used one of each type of story in her research as well (Booth, personal communication 2006). The stories have been found to reliably discriminate children with known mental-state identification deficits, such as those with ASDs, from typically developing children [37]. The child’s answers for each story were scored on a 3-point scale with 0 being awarded for an incorrect answer, 1 point for a partially-correct answer, and 2 points for a fully-correct answer. The participant’s total score (i.e., the sum of the score for each story, out of 24) was used for analyses. One quarter of responses were coded by another experimenter who was blind to the participant’s performance on other measures (κ = .81).

#### Verbal Ability

To assess verbal ability, the Verbal Comprehension test of the Woodcock-Johnson III (WJ-III) [38] was administered. Despite its label, the Verbal Comprehension test involves both language comprehension (receptive language) and language production. This test is comprised of four subtests measuring expressive lexical knowledge (e.g., Can you tell me what [picture] is called?), synonyms (e.g., Tell me another word for _____), antonyms (e.g., What is the opposite of _____?), and analogies (e.g., Finish the sentence – A is to B as C is to _____). Verbal Mental Age is computed based on the participant’s performance on the four subtests.

### Procedure

Participants were first administered either the two Eyes Tasks (both the *ET* and *GET*) or the Strange Stories (for typically-developing students only), counterbalanced. The *GET* always preceded the *ET* so that participants could not simply echo terms provided in the forced-choice format when completing the *GET*, as previous work has shown that performance on forced-choice tasks predicts performance on open-ended tasks when administered first [39]. Participants were then given the Verbal Comprehension test of the WJ-III.

## Results: Study 2

### Discriminant Validity Analyses

#### Comparing the ET vs. the GET

To make comparisons between the *ET* and the *GET*, scores were transformed to proportion scores. The *ET* was significantly easier than the *GET* as it was in Study 1, though differently, the 10-item *GET* was also significantly more difficult than the *ET*. These differences were larger for the older sample than the younger group (which was more in line with the age range in Study 1). The two tasks were not correlated, *r = *−.097, *n.s.* [*r = *–.032, *n.s.*; 10-item *GET*]. See Table 1 for a full comparison. The fact that the two are not more highly related is consistent with our claim that changing the nature of the response format changes the very nature of the task and what it measures.

#### Relationships with Verbal Ability and Age

To determine the relative relationship between the *ET* and the *GET* with verbal ability, both scores were included in a linear regression predicting the child’s verbal mental age. This was done to allow direct comparisons between the *ET* and *GET*. Results indicated that the *ET*, β = .471, *p*<.001 [β = .450, *p*<.001; 10-item *GET*] was a far better predictor of verbal mental age than the *GET*, β = –.136, *p* = .167 [β = –.237, *p* = .014; 10-item *GET*]. These results reveal that the *ET* is more strongly correlated with verbal ability than the *GET*.

Verbal ability and age were highly correlated, as expected, *r = *.661, *p<*.001, and thus the relationships between age and the Eyes Tasks were nearly identical to those with verbal ability. The *ET* was over five times more predictive of age, β_ET_ = .366, *p* = .001 versus β_GET_ = .069, *p* = .501 [β_ET_ = .369, *p*<.001 versus β_GET_ = –.131, *p* = .202; 10-item *GET*]. Interestingly, when verbal ability was included in the models, the beta for the *GET* became nearly significant, β = .157, *p* = .060, whereas the beta for the *ET* became non-significant, β = .061, *p = *.508, revealing that, when verbal ability is controlled for, the *GET* is correlated with age whereas the *ET* is not. The relationship between the *ET* and age is dependent upon changes in verbal ability with age, whereas the *GET* is not.

#### Relationships with Happé’s Strange Stories

Linear regressions were used to compare the relationships of the *ET* and *GET* with another measure of cognitive perspective-taking, *Happé’s Strange Stories*. The two Eyes Tasks were included in the same regression predicting the *Strange Stories*. The *ET* was nearly nine times as predictive of performance on the *Strange Stories* than the *GET*, β_ET_ = .332, *p* = .002 versus β_GET_ = –.037, *p* = .725 [β_ET_ = .327, *p* = .002 versus β_GET_ = –.043, *p* = .679; 10-item *GET*]; however, when verbal ability was controlled for, neither task was a significant predictor, β_ET_ = .138, *p* = .215 versus β_GET_ = .019, *p* = .847 [β_ET_ = .135, *p* = .220 versus β_GET_ = .058, *p* = .568; 10-item *GET*].

### Group Comparisons Analyses

#### Preliminary Analyses

As the stimuli sets used for the ET and GET were counterbalanced for the learning disordered group, results for the ET and the GET were first compared by stimuli set. There were no differences in performance between sets on either the ET, t(52) = 1.476, p = .146, Cohen’s d = .41, or the GET, t(52) = .594, p = .555, Cohen’s d = .16. Due to the lack of differences by stimuli set, the data were combined for all further analyses.

To determine if the participants had verbal deficits relative to the typically-developing participants, independent group t-tests on age and verbal ability were conducted. Results showed no significant difference in verbal ability, *t(137) = *−.846, *n.s., Cohen’s d = *.14 (M_diff_ = 0.39 yrs), despite a significant difference in mean age, *t(137) = *−10.729, *p<*.001, *Cohen’s d = *1.83, with the learning-disordered participants being significantly older than the typically-developing participants (M_diff_ = 3.05 yrs). Therefore, the learning-disordered participants presented with a verbal deficit compared to the typically-developing participants, relative to their mean age.

#### Comparing the ET and the GET

**Figure 1:**
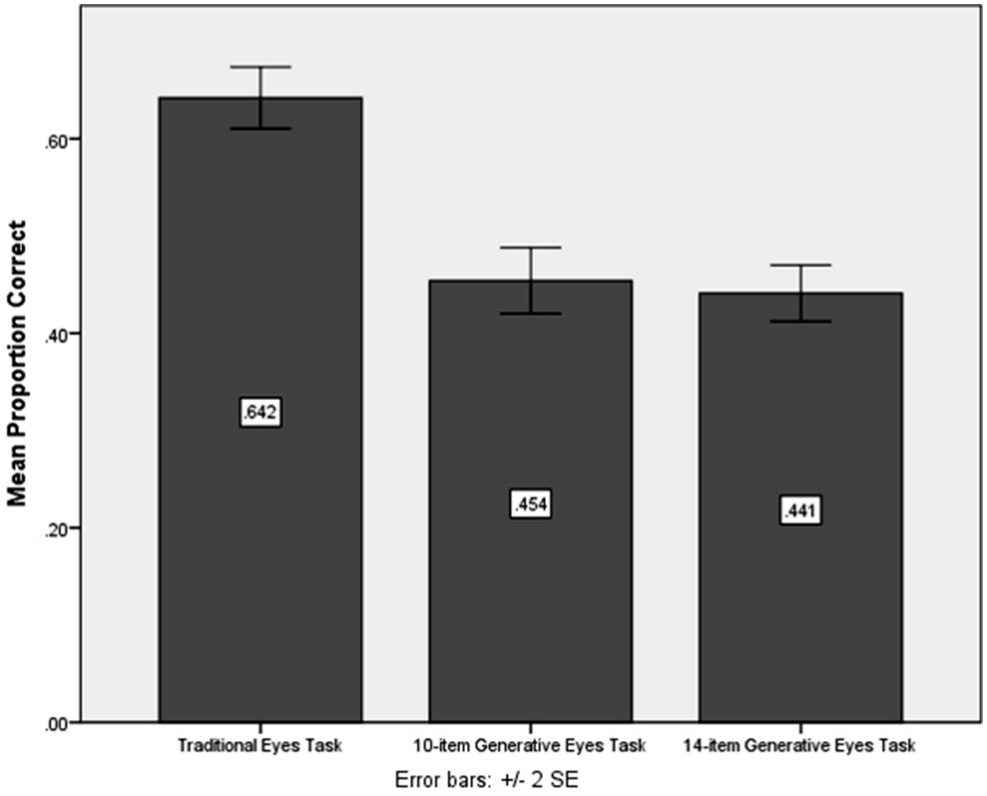
Comparison of performance on the *ET* and *GET* in the learning-disordered group (Study 2). Children in the learning-disordered group who were identified as having difficulty with social-emotional skills do not seem to show a floor effect with respect to the Generative Eyes Task (*GET*), a concern when testing a clinical population. However, compared to the traditional Eyes Task (*ET*), the *GET* remains more difficult for this group as a whole as evidenced by statistically significant differences in performance between the two versions of the *GET* and the *ET*. Error bars represent 2 SDs of standard error.

**Figure 2:**
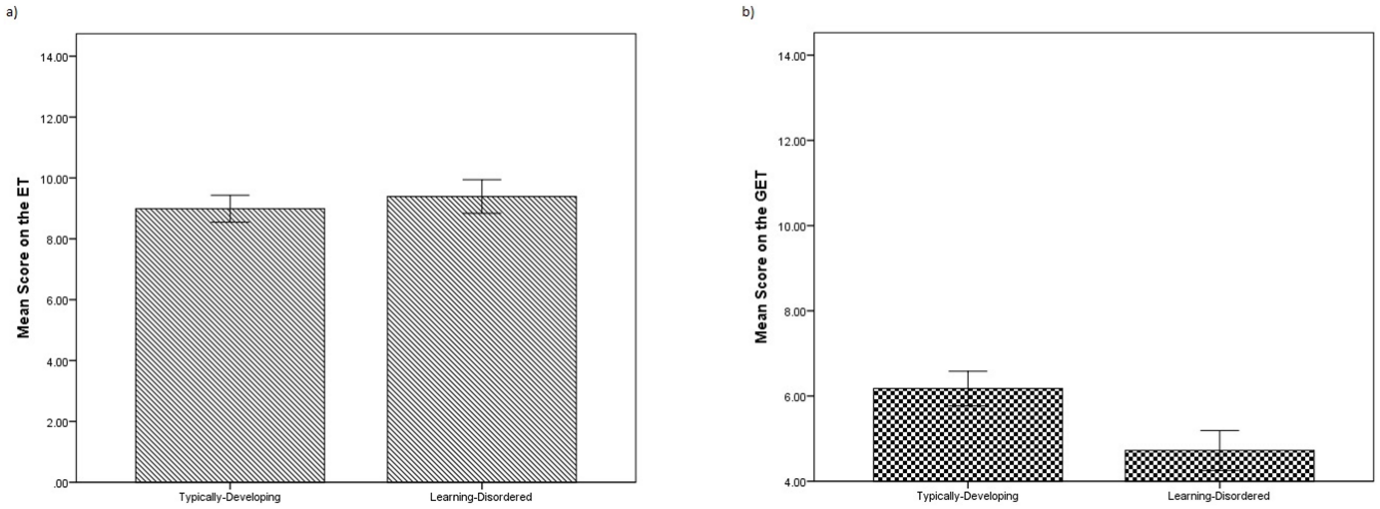
Comparison of performance on the *ET* (2a) and *GET* (2b) between the learning-disordered or typically-developing groups (Study 2). Comparisons between the two groups on the Eyes Task (*ET*; Figure S2a) and the Generative Eyes Task (*GET*; Figure S2b) show that although there is no difference in performance on the *ET*, there is a difference on the *GET*. Notably, the two groups were comparable in verbal ability (despite the learning-disordered group being older), suggesting that verbal ability is key to the *ET*, but not the *GET*. The difference between the two groups on the *GET* was statistically significant at p<.05. Error bars represent 2 SDs of standard error.

Performance on the *GET* was significantly worse than performance on the *ET*, *t(53) = *−13.743, *p<*.001, *Cohen’s d = *3.78, but not so much so that it resulted in a floor effect (see Figure 1). When the two measures were correlated with verbal ability, the *ET* was significantly related, *r = *.284, *p<*.05, but not the *GET*, *r = *.159, *n.s.*, implying that the *ET* was recruiting verbal ability in the clinical sample, as was also true of the typically-developing sample. Furthermore, when performances on the *ET* and *GET* were compared for the learning-disordered and typically-developing samples, there were no group differences on the *ET*, *t(137) = *−1.129, *n.s., Cohen’s d = *.19. However, there was a significant difference between the two groups on the *GET*, *t(137) = *4.615, *p<*.001, *Cohen’s d = *.79, with typically-developing participants performing significantly better than the clinical sample (see Figure 2).

#### Predicting Group Membership

To determine if the group differences could be predicted by the *ET* or *GET,* a Logistical Regression analysis was run predicting group membership (i.e., clinical versus typically-developing). In step 1, age and verbal ability were entered to control for their effects. In step 2, scores for the *ET* and *GET* were entered. Although age (odds-ratio = 1.149 [CI: 1.093,1.208]) and verbal ability (odds-ratio = .956 [CI: .934,.979]) were both significant predictors of group membership, performance on the *GET* was the most significant predictor, with a unit decrease in performance increasing the odds of being in the learning-disordered group by 87% (odds-ratio = .533[CI: .382,.743] with typically-developing as the baseline group). Importantly, the model was able to correctly classify 88.5% of individuals into their respective group–clinical or typically-developing. There was no such significance for the *ET* (odds-ratio = .980[CI: .704,1.366]).

## Discussion: Study 2

The goals of this study were first to compare and contrast a shortened forced-choice version of the Eyes Task with an equally shortened open-ended version. Furthermore, to examine their relationships to verbal ability and another widely-used open-ended perspective-taking task (*Happé’s Strange Stories*) believed to rely predominantly on cognitive rather than affective components of perspective-taking. A final goal was to determine how the measures fare in discriminating typically-developing children from those who have moderate deficits in affective perspective-taking.

When comparing the shortened versions of the *ET* and *GET*, the results are consistent with our prediction that the *GET* is unrelated to cognitive aspects of perspective-taking (at least as measured by the *Strange Stories* task). The *ET*, that we hypothesized to be related to cognitive abilities, was more strongly associated with verbal ability and *Happé’s Strange Stories*, as a measure of cognitive perspective-taking. However, when verbal ability was controlled for, the relationship with *Happé’s Strange Stories* became non-significant, suggesting that the primary source of shared variance between the *ET* and the *Strange Stories* task was their shared reliance on verbal ability. In contrast, the *GET* had no relationship with verbal ability or *Happé’s Strange Stories*. This was particularly important as one of the goals was to create a task that does not rely on advanced verbal ability. After all, a high level of verbal ability is not *necessary* for recognizing others’ emotions and responding appropriately, therefore, tasks that require a high level of verbal ability may fail to accurately capture the affective perspective taking skills of individuals without that level of proficiency (e.g., young children, individuals with ASDs and other disorders linked to verbal deficits). The significant correlation between age and the *GET* (when verbal ability is controlled for) suggests that the *GET* is identifying moderate improvements in emotion recognition abilities that are unrelated to one’s vocabulary knowledge of specific emotion labels. The *ET*, in contrast, appears to be primarily identifying improvements in vocabulary instead of age-related changes in perspective-taking performance per se.

Regarding the ability of the individual measures to discriminate between a clinical group of children with learning disorders and typically-developing children, results showed not only that those with learning disorders performed worse on the *GET* relative to the typically-developing sample, but also that the *GET* was a significant predictor in classifying individuals into their respective clinical or typically-developing group, in line with earlier work on learning disorders and emotion recognition [35]. The *ET*, on the other hand, did not detect any differences between the clinical and typically-developing groups. The *GET* was a more difficult task than the *ET*, suggesting that either the *ET* inflates performance (e.g., by allowing for process of elimination) or is assessing something other than emotion recognition per se. As in the previous results, performance in the *ET* was related to verbal ability whereas performance on the *GET* was not; this may explain the lack of group differences on the *ET* as the two groups did not differ in their verbal ability.

Importantly, the *GET* was not so difficult that it resulted in a floor effect, indicating it may be successfully used to test emotion recognition even in a clinical sample with low verbal ability and learning disabilities. The failure to find any significant differences between the stimuli sets (i.e., the first half of the stimuli vs. the second half of the stimuli) used in the *ET* and *GET* demonstrates that the results do not stem from differences in the specific images used, but rather reflect inherent differences in the forced-choice versus open-ended nature of the tasks. Additionally, variability in the clinical group was similar to that of the typically-developing group for both the *GET* (σ_C_^2^ = 3.48, σ_T_^2^ = 2.96) and the *ET* (σ_C_^2^ = 4.18, σ_T_^2^ = 4.13); therefore, it is unlikely that the results are due to any differences in variability between the learning-disordered and typically-developing groups.

## General Discussion

The current studies compared the widely-used *Reading the Mind in the Eyes* task [1]–[3] with a modified open-ended version in their a) relationship to verbal ability, b) relationship to dispositional empathy, c) relationship to a measure of cognitive perspective taking that also used an open-ended format, and d) ability to predict group membership between those with and without social-emotional deficits. Following the logic underlying the plethora of research discriminating the processes tapped by recognition (i.e., forced-choice) versus recall (i.e., open-ended) memory tasks [11]–[12], we hypothesized that the *GET* would be a more powerful and specific measure of emotion recognition.

Results were in line with this hypothesis. First, the *GET* was *un*related to verbal ability (Study 2) and was a better predictor of dispositional empathy (Study 1), a well-established correlate of emotion recognition, than the *ET*. Furthermore, the *GET* differentiated individuals from a clinical population from those in a non-clinical one (Study 2). Second, we found that the forced-choice nature of the *ET* taps processes (e.g., verbal ability) that are not specific to perspective-taking or part of perspective-taking in naturalistic situations. Furthermore, the *ET*, unlike the *GET*, is amenable to compensatory strategies such as ‘process of elimination’ that could inflate participants’ scores and mask subtle deficits in emotion recognition and perspective taking. We urge researchers to consider these findings when deciding which response format (open-ended vs. forced-choice) is the most appropriate for use with their population of interest and their specific research questions. We suggest that the *GET* may be more appropriate when researchers are specifically interested in emotion recognition or affective perspective taking (as opposed to cognitive perspective taking or general perspective taking abilities) and when working with populations with limited verbal abilities (e.g., very young children or those with learning disorders or verbal deficits).

The open-ended nature of the *GET* also allows young children, and others with low verbal ability, to provide responses that capture emotional significance using age- or ability-appropriate language, opening the door for researchers to test emotion recognition in younger and broader populations than the *ET*. The *GET* was unrelated to verbal ability, which some may view as counterintuitive due to the verbal demands of generating words. We believe, however, that all participants reached the minimum verbal ability required to simply speak about emotions. Once this minimum is reached, there is no reason to believe that verbal ability would influence the score on the *GET* due to the valence-based nature of the coding system. A child who responds with “Happy” to a positive-valence item would be as correct as a child with a higher verbal ability who may identify it as “Ecstatic” or “Joyful”. As such, we would only expect verbal ability to predict differences in terms of nuanced language used within a particular valence, but not performance per se. In turn this then allows for a more thorough examination of the developmental trajectory of perspective-taking using the same measure across ages. Future research should also address whether the *GET* is appropriate for predicting other outcomes (e.g., aggressive tendencies) and whether or not changing the *ET* to same-valence options for the four choices or to offer four different valence terms as the four options would lead to greater efficacy as an affective perspective-taking task.

We are not the first to suggest that the verbal load of the *ET* may not be suitable for younger children. Peterson and Slaughter [40] simplified the *ET*, utilizing 2 items with a lower verbal load and administered it to individuals aged 3 to adulthood. They found that the simplified version was correlated with false-belief understanding in children, a well-known measure of a milestone in cognitive perspective-taking. It seems that the *ET* provides stimuli that are beneficial to examining at least some aspects of perspective-taking, but that the current methodology may not be ideal for examining these abilities in *all* children due to the verbal demands. The *GET*, with the focus on the perceptual recognition of the expression (rather than a combination of expression recognition and word recognition), provides a more accurate assessment of a child’s ability to read perceptual cues to emotions. The latter is more representative of real-life emotion recognition situations and therefore also a more ecologically valid task.

In considering potential limitations of the present findings, one might question whether the results merely reflect a more ‘lenient’ coding of the *GET* compared to the *ET*; we discredit this idea for the following reasons. First, the categorical coding of the *GET* parallels the requirements of the *ET* because the four forced-choice terms provided in each *ET* item rarely if ever include a same-category alternative when the *correct* response was emotional in nature (see Appendix S1). Second, results across the three studies suggest that participants found the *GET* task *harder* than the *ET*. If the coding were more lenient, we would expect to find participants performing much better on the *GET*.

A second concern could be that the *GET* did not correlate with age, despite the somewhat larger age-range tested (outside of the marginal association in Study 2). One might wonder whether the lack of correlation with age implies a limitation of the measure, however, we argue that it is not surprising that there are *not* significant age-related changes in the sample measured. Neurological structures implicated in emotion recognition have been found to be developed by the age of 3–4 [41], suggesting future developmental changes should be small to moderate, a prospect that is supported in the literature. Consider for example, research looking at facial emotion recognition in children aged 5 to 11 and adults that found very little developmental change in the ability to identify the basic emotions (happiness, anger, and sadness) between 5-year-olds and adults (only fear showed a relatively large increase, from 82% correct in 5-year-olds to 97% in adults) [42]. Thus, the marginal association in Study 2 likely reflects this small development that has been highlighted elsewhere, but any larger association would be unexpected.

It is important to acknowledge that the *GET* will not always be preferable to the *ET*; each has their advantages and disadvantages depending on the research objectives. One advantage of the forced-choice version is its known utility in distinguishing between those with and without ASDs. The *GET* may well serve this same function; it has not been assessed herein and thus we cannot speak to the possibility directly. We do know, however, that the *GET* distinguishes between those with mild-moderate social-emotional deficits and typically-developing children. More research will allow for a better understanding of which age groups and populations are best served by the use of the *GET* as compared to the *ET.* Notably, there is considerable evidence in the literature showing that the forced-choice task is a decent measure of perspective-taking in general, a finding supported by the statistical comparison between the two tasks [29]. The *GET*, on the other hand, appears to be advantageous for the purpose of specifically identifying emotion recognition deficits, for predicting empathy, and for differentiating between learning-disordered individuals and typically-developing individuals.

The results herein provide a strong starting point for the utilization of a new method for assessing emotion recognition. The current research provides important steps toward increasing the utility of the measurement tools that are available to researchers and improving the study of emotion recognition and its distinction from cognitive perspective-taking. Although there is still a need for further research (e.g., does the *GET* provide a better predictor than the *ET* of other outcomes such as aggressive tendencies?), we hope the current findings encourage researchers to consider the important lessons from the literature contrasting recall and recognition memory tasks and bear in mind those lessons, and other considerations discussed above, when designing their studies and drawing conclusions from open-ended versus forced-choice emotion recognition and perspective-taking tasks.

## Supporting Information

Appendix S1 Coding scheme and examples of correct answers for the *ET* and *GET*.
(DOCX)

## References

[1] Baron-Cohen S, Jolliffe T, Mortimore C, Robertson M (1997) Another advanced test of social perspective-taking: Evidence from very high functioning adults with autism or Asperger’s syndrome. J Child Psychol Psychiatry 38:813–822. 9363580 10.1111/j.1469-7610.1997.tb01599.x

[2] Baron-Cohen S, Wheelwright S, Hill J, Raste Y, Plumb I (2001) The “Reading the Mind in the Eyes” test revised version: A study with normal adults, and adults with Asperger’s syndrome or high-functioning autism. J Child Psychol Psychiatry 42:241–251. 11280420

[3] Baron-Cohen S, Wheelwright S, Spong A, Scahill V, Lawson J (2001) Are intuitive physics and intuitive psychology independent? A test with children with Asperger’s syndrome. J Dev Learn Disord 5:47–78.

[4] Craig JS, Hatton C, Craig FB, Bentall RP (2004) Persecutory beliefs, attributions and theory of mind: Comparison of patients with paranoid delusions, Asperger's syndrome and healthy controls. Schizophr Res 69:29–33. 15145468 10.1016/S0920-9964(03)00154-3

[5] Lee L, Harkness K, Sabbagh M, Jacobson J (2005) Mental state decoding abilities in clinical depression, J Affect Disord. 86:247–258. 10.1016/j.jad.2005.02.007 15935244

[6] Sharp C (2007) Theory of mind and conduct problems in children: Deficits in reading the “emotions of the eyes”. Cogn Emot 22:1149–1158.

[7] Dodge KA (1980) Social cognition and children’s aggressive behaviour. Child Dev 51:162–170. 7363732

[8] Hughes C, Dunn J, White A (1998) Trick or treat? Uneven understanding of mind and emotion and executive dysfunction in ‘hard-to-manage’ preschoolers. J Child Psychol Psychiatry 39:981–994. 9804031

[9] Izard C, Fine S, Schultz D, Mostow A, Ackerman B, et al (2001) Emotion knowledge as a predictor of social behaviour and academic competence in children at risk. Psychol Sci 12:18–23. 11294223 10.1111/1467-9280.00304

[10] Knafo A, Steinberg T, Goldner I (2011) Children’s low affective perspective-taking ability is associated with low self-initiated pro-sociality. Emotion 11:194–198. 21401240 10.1037/a0021240

[11] Breen EK (1993) Recall and recognition memory in Parkinson’s disease. Cortex 29:91–102. 8472561 10.1016/s0010-9452(13)80214-6

[12] Calev A (1984) Recall and recognition in chronic nondemented schizophrenics. J Abnorm Psychol 93:172–177. 6725750 10.1037//0021-843x.93.2.172

[13] Gordon HL, Baird AA, End A (2004) Functional differences among those high and low on a tendency measure of psychopathy. Biol Psychiatry 56:516–521. 15450788 10.1016/j.biopsych.2004.06.030

[14] Harris PL (2006) Social Cognition. In D. Kuhn et al.(Eds), Handbook of child psychology: Volume 2, Cognition, perception, and language (6th ed.). Hoboken, NJ, US: John Wiley & Sons Inc 811–858.

[15] Happé FGE (1995) The role of age and verbal ability in the theory of mind task performance of subjects with Autism. Child Dev 66:842–855. 7789204

[16] Stone V, Baron-Cohen S, Young A, Calder A (1998) Patients with amygdalectomy show impairments in theory of mind. Cambridge: University of Cambridge.

[17] Gregory C, Lough S, Stone V, Erzinclioglu S, Martin L, et al (2002) Theory of mind in patients with frontal variant frontotemporal dementia and Alzheimer’s disease: theoretical and practical implications. Brain 125:752–764. 11912109 10.1093/brain/awf079

[18] Richell RA, Mitchell DGV, Newman C, Leonard A, Baron-Cohen S, et al (2003) Social perspective-taking and psychopathy: Can psychopathic individuals read the ‘language of the eyes’? Neuropsychologia 41:523–526. 12559146 10.1016/s0028-3932(02)00175-6

[19] Tonks J, Williams WH, Frampton I, Yates P, Slater A (2007) Assessing emotion recognition in 9–15 year olds: Preliminary analysis of abilities in reading emotion from faces, voices and eyes. Brain Inj 21:623–629. 17577713 10.1080/02699050701426865

[20] Tonks J, Williams WH, Frampton I, Yates P, Slater A (2007) Reading emotions after child brain injury: a comparison between children with brain injury and non-injured controls. Brain Inj 21:731–739. 17653947 10.1080/02699050701426899

[21] Glass SJ, Newman JP (2006) Recognition of Facial Affect in Psychopathic Offenders. J Abnorm Psychol 115:815–820. 17100539 10.1037/0021-843X.115.4.815

[22] Dolan M, Fullam R. (2006) Face affect recognition deficits in personality-disordered offenders: association with psychopathy. Psychol Med 36:1563–1569. 16893483 10.1017/S0033291706008634

[23] Blair RJR, Colledge E, Murray L, Mitchell DGV (2001) A selective impairment in the processing of sad and fearful expressions in children with psychopathic tendencies. J Abnorm Child Psychol 29:491–498. 11761283 10.1023/a:1012225108281

[24] Widen SC, Russell JA (2003) A closer look at preschoolers’ freely produced labels for facial expressions. Dev Psychol 39:114–128. 12518813 10.1037//0012-1649.39.1.114

[25] Lane RD, Sechrest L, Reidel R, Weldon V, Kaszniak A, et al (1996) Impaired verbal and nonverbal emotion recognition in alexithymia. Psychosom Med 58:203–210. 8771618 10.1097/00006842-199605000-00002

[26] Pollak SD, Cicchetti D, Hornung K, Reed A (2000) Recognizing emotion in faces: developmental effects of child abuse and neglect. Dev Psychol 36:679–688. 10976606 10.1037/0012-1649.36.5.679

[27] Ribordy SC, Camras LA, Stefani R, Spaccarelli S (1988) Vignettes for emotion recognitions research and affective therapy with children. J Clin Child Psychol 17:322–325.

[28] Carey J, Cassels TG (2013) Comparing two forms of a childhood perspective-taking measure using CFA and IRT. Psychol Assess 25:879–892. 23647048 10.1037/a0032641

[29] Batson CD, Early S, Salvarani G (1997) Perspective-taking: Imagining how another feels versus imagining how you would feel. Pers Soc Psychol Bull 23:751–758.

[30] Hinnant JB, O’Brien M (2007) Cognitive and emotional control and perspective-taking and their relations to empathy in 5-year-old children. J Genet Psychol 168:301–322. 18200891 10.3200/GNTP.168.3.301-322

[31] Hooker CI, Verosky SC, Germine LT, Knight RT, D’Esposito M (2008) Mentalizing about emotion and its relationship to empathy. Soc Cog Affect Neurosci 3:204–217. 10.1093/scan/nsn019 PMC2566770 19015112

[32] Bryant BK (1982) An index of empathy for children and adolescents. Child Dev 53:413–425.

[33] Spearman C (1904) The proof and measurement of association between two things. Am J Psychol 15:72–101. 3322052

[34] Bengtsson H, Johnson L (1992) Perspective taking, empathy, and prosocial behavior in late childhood. Child Study 22:11–21.

[35] Owen A, Browning M, Jones RSP (2001) Emotion recognition in adults with mild-moderate learning disabilities. J Learn Disabil 5:267–281.

[36] American Psychiatric Association (2000) Diagnostic and Statistical Manual of Mental Disorders - Fourth Edition – Text Revised. Washington, DC: American Psychiatric Publishing, Inc.

[37] Happé FGE (1994) An advanced test of social perspective-taking: Understanding the story characters’ thoughts and feelings by able autistic, mentally handicapped, and normal children and adults. J Autism Dev Disord 24:129–154. 8040158 10.1007/BF02172093

[38] Woodcock RW, McGrew KS, Mather N (2001) Woodcock-Johnson III Brief Intellectual Assessment (WJ-III BIA). Itasca: Riverside Publishing.

[39] Harrigan JA (1984) The effects of task order on children’s identification of facial expressions. Motiv Emot 8:157–169.

[40] Peterson CC, Slaughter V (2009) Theory of mind (TOM) in children with autism or typical development: Links between eye-reading and false belief understanding. Res Autism Spectr Disord 3:462–473.

[41] Nelson CA (2001) The development and neural bases of face recognition. Infant Child Dev 10:3–18.

[42] Durand K, Gallay M, Seigneuric A, Robichon F, Beaudouin JY (2007) The development of facial emotion recognition: The role of configural information. J Exp Child Psychol 97:14–27. 17291524 10.1016/j.jecp.2006.12.001

