# Supplementary material for: Comparisons of an Open-Ended vs. Forced-Choice ‘Mind Reading’ Task: Implications for Measuring Perspective-Taking and Emotion Recognition
Source: PLoS One. 2014 Dec 4;9(12):e93653. doi: 10.1371/journal.pone.0093653 (PMC4256375; doi:10.1371/journal.pone.0093653)
Supplement: Appendix S1 — Coding scheme and examples of correct answers for the ET and GET. (DOCX) [file pone.0093653.s001.docx]

**Appendix S1. Correct Answers for the Eyes Task and the Generative Eyes Task**

| Item | Term 1 | Term 2 | Term 3 | Term 4 | Correct Category (GET) | An Example of a Correct Answer (GET) |
| --- | --- | --- | --- | --- | --- | --- |
| 1 | Hate | Surprised | **Kind** | Cross | Positive | Happy |
| 2 | Unkind | Cross | Surprised | **Sad** | Negative | Unhappy |
| 3 | **Friendly** | Sad | Surprised | Worried | Positive | Happy |
| 4 | Relaxed | **Upset** | Surprised | Excited | Negative | Sad |
| 5 | Feeling sorry | **Making somebody do something** | Joking | Relaxed | Neutral | Serious |
| 6 | Hate | Unkind | **Worried** | Bored | Negative | Nervous |
| 7 | Feeling sorry | Bored | **Interested** | Joking | Positive | Surprised |
| 8 | **Remembering** | Happy | Friendly | Angry | Neutral | Thoughtful |
| 9 | Annoyed | Hate | Surprised | **Thinking about something** | Neutral | Confused |
| 10 | Kind | Shy | **Not believing** | Sad | Negative | Weary |
| 11 | Bossy | **Hoping** | Angry | Disgusted | Positive | Grateful |
| 12 | Confused | Joking | Sad | **Serious** | Neutral | Focused |
| 13 | **Thinking about something** | Upset | Excited | Happy | Neutral | Curious |
| 14 | Happy | **Thinking about something** | Excited | Kind | Neutral | Normal |
| 15 | **Not believing** | Friendly | Wanting to play | Relaxed | Negative | Anxious |
| 16 | **Made up her mind** | Joking | Surprised | Bored | Neutral | Serious |
| 17 | Angry | Friendly | Unkind | **A bit worried** | Negative | Sad |
| 18 | **Thinking about something sad** | Angry | Bossy | Friendly | Negative | Lonely |
| 19 | Angry | Daydreaming | Sad | **Interested** | Positive | Happy |
| 20 | Kind | Surprised | **Not pleased** | Excited | Hostile | Mad |
| 21 | **Interested** | Joking | Relaxed | Happy | Positive | Sly |
| 22 | Playful | Kind | Surprised | **Thinking about something** | Neutral | Normal |
| 23 | Surprised | **Sure about something** | Joking | Happy | Neutral | Determined |
| 24 | **Serious** | Ashamed | Confused | Surprised | Neutral | Obsessed |
| 25 | Shy | Guilty | Daydreaming | **Worried** | Negative | Depressed |
| 26 | Joking | Relaxed | **Nervous** | Sorry | Negative | Scared |
| 27 | Ashamed | Excited | **Not believing** | Pleased | Negative | Unhappy |
| 28 | Disgust | Hate | **Happy** | Bored | Positive | Calm |

Note: The four terms are those included in the *ET* with the correct answer is in bold. For the full stimuli and package, please visit The Autism Research Centre at the University of Cambridge. The *GET* correct valence is listed along with one example of a correct answer that was given by participants in the studies
